# Exposure to bacterial endotoxin generates a distinct strain of α-synuclein fibril

**DOI:** 10.1038/srep30891

**Published:** 2016-08-04

**Authors:** Changyoun Kim, Guohua Lv, Jun Sung Lee, Byung Chul Jung, Masami Masuda-Suzukake, Chul-Suk Hong, Elvira Valera, He-Jin Lee, Seung R. Paik, Masato Hasegawa, Eliezer Masliah, David Eliezer, Seung-Jae Lee

**Affiliations:** 1 Department of Biomedical Sciences, Neuroscience Research Institute, Seoul National University College of Medicine, Seoul, Korea; 2 Departments of Neurosciences and Pathology, School of Medicine, University of California, San Diego, La Jolla, CA, USA; 3 Department of Biochemistry, Weill Cornell Medical College, NY, USA; 4 Department of Biomedical Laboratory Science, College of Health Science, Yonsei University, Wonju, Korea; 5 Department of Neuropathology and Cell Biology, Tokyo Metropolitan Institute of Medical Science, Tokyo, Japan; 6 School of Chemical and Biological Engineering, College of Engineering, Seoul National University, Seoul, Korea; 7 Department of Anatomy, School of Medicine, Konkuk University, Seoul, Korea

## Abstract

A single amyloidogenic protein is implicated in multiple neurological diseases and capable of generating a number of aggregate “strains” with distinct structures. Among the amyloidogenic proteins, α-synuclein generates multiple patterns of proteinopathies in a group of diseases, such as Parkinson disease (PD), dementia with Lewy bodies (DLB), and multiple system atrophy (MSA). However, the link between specific conformations and distinct pathologies, the key concept of the strain hypothesis, remains elusive. Here we show that in the presence of bacterial endotoxin, lipopolysaccharide (LPS), α-synuclein generated a self-renewable, structurally distinct fibril strain that consistently induced specific patterns of synucleinopathies in mice. These results suggest that amyloid fibrils with self-renewable structures cause distinct types of proteinopathies despite the identical primary structure and that exposure to exogenous pathogens may contribute to the diversity of synucleinopathies.

Deposition of specific protein aggregates is a key pathological feature of prion diseases and various neurodegenerative diseases^1^. Aggregates come in different flavors with distinct conformations. In prion diseases, different types of prion aggregates represent different disease phenotypes, such as incubation times, symptoms, and pathology, hence being referred to as strains^1^,^2^,^3^. These aggregates are composed of specific conformational elements that grow single dimensionally^2^. Growth of the aggregates involves conversion of monomeric proteins to the aggregates, and by doing so, the specific conformations propagate. This “seeded” aggregation concept is thought to be the underlying mechanism for prion propagation and prion strains. Strikingly, the seeded aggregation is a shared characteristic among the aggregated proteins that are linked to neurodegenerative diseases, such as amyloid-β, α-synuclein, and tau^4^. Recent findings suggest that aggregates of these proteins spread through the brain cells in a prion-like manner, which might explain the mechanism of disease progression^5^.

Parkinson’s disease (PD), dementia with Lewy bodies (DLB), and multiple system atrophy (MSA) are age-related, progressive, devastating neurodegenerative disorders that are distinct but overlap both clinically and pathologically^6^. Deposition in neurons and glial cells of clumps of a neuronal protein known as α-synuclein is the shared characteristic. What sets these diseases apart is the anatomical patterns and cell type distribution of α-synuclein deposition. Deposition of this protein happens early in the progression of the disease at the synaptic connections and later on spreads to the neuronal cell bodies forming Lewy bodies (LBs) and Lewy neurites (LNs). Both PD and DLB are thought to be different in the patterns of anatomical spreading of small α-synuclein aggregates in synapses as well as in LBs and LNs during the disease progression. Furthermore, even in the same disease the patterns of α-synuclein deposition, as well as clinical symptoms, varies depending on the individual cases. However, the origin of heterogeneity in α-synuclein deposition, hence the clinical manifestation, remains unknown.

Like many other amyloidogenic proteins, α-synuclein can produce distinct fibril “strains” that are capable of self-renewal through templated conformational conversion *in vitro*^7^,^8^. Although recent a study has reported that different α-synuclein fibril subtypes exhibited different neurotoxic properties and induced different patterns of aggregate deposition^9^, the origination of different strains remains largely unknown.

Several environmental toxins have been implicated in PD^10^. Viral and bacterial infections have also been suggested to be the cause of PD^11^,^12^,^13^. In animals, administration of a bacterial endotoxin, lipopolysaccharides (LPS), caused dopaminergic degeneration and motor deficits^14^,^15^. Herein, to assess the potential environmental influences on PD pathology and heterogeneity, we generated two structurally distinct α-synuclein fibrils in the presence and absence of LPS and demonstrated the strain-like behaviors of these fibrils *in vivo*.

## Results

### Bacterial endotoxins induce the formation of α-synuclein fibrils with distinct structures

**Figure 1:**
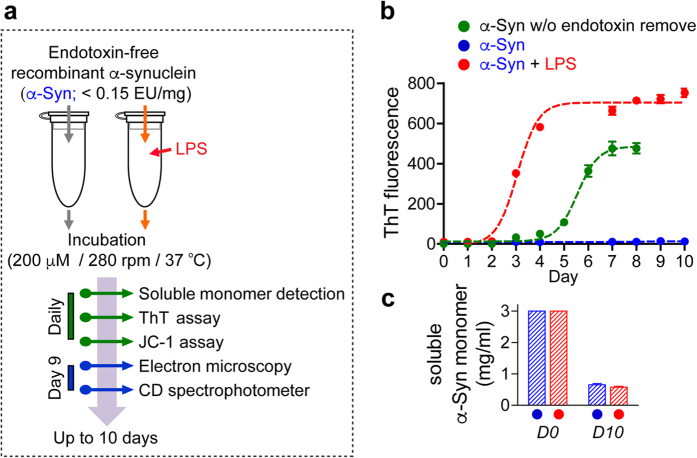
Generation of two distinct α-synuclein fibril polymorphs. (**a**) Experimental scheme. Endotoxin-free recombinant human α-synuclein was incubated in the presence or absence of LPS (100 μg/ml) for 10 days, and analyzed by indicated analysis. Data are representative of three independent experiments. (**b,c**) ThT fluorescence analysis (**b**) and monomer consumptions analysis (**c**) were performed with daily samples.

To determine the effects of bacterial endotoxins on α-synuclein fibrillation, we prepared endotoxin-free α-synuclein with endotoxin concentrations of less than 0.015 units per mg protein^16^. The endotoxin-free α-synuclein was incubated with agitation and fibrillation was analyzed with various methods (Fig. 1a). When the kinetics of fibrillation was analyzed with Thioflavin T (ThT) binding assay, endotoxin-free α-synuclein exhibited only a weak ThT binding for 10 days. In contrast, the α-synuclein preparation without endotoxin removal generated a strong ThT fluorescence with the lag phase of 4 days (Fig. 1b). When lipopolysaccharide (LPS), a common endotoxin of gram-negative bacteria, was added to the endotoxin-free α-synuclein, fibrillation with a strong ThT reactivity reached the equilibrium phase within 5 days with the lag phase of less than 2 days (Fig. 1b), suggesting that the presence of endotoxins, such as LPS, has profound effects on fibrillation of α-synuclein.

To examine whether the ThT fluorescence represented the amounts of fibrils generated, we measured monomer consumption. In contrast to the differences in ThT fluorescence, both LPS(−)α-synuclein and LPS(+)α-synuclein exhibited the same extent of monomer loss at day 10 (Fig. 1c), indicating that LPS(−)α-synuclein formed aggregates with weak ThT binding. The kinetic studies exhibited a strong correlation between ThT fluorescence and monomer loss (Supplementary Fig. 1a,b). ThT fluorescence was not generated by LPS alone (Supplementary Fig. 1c).

**Figure 2:**
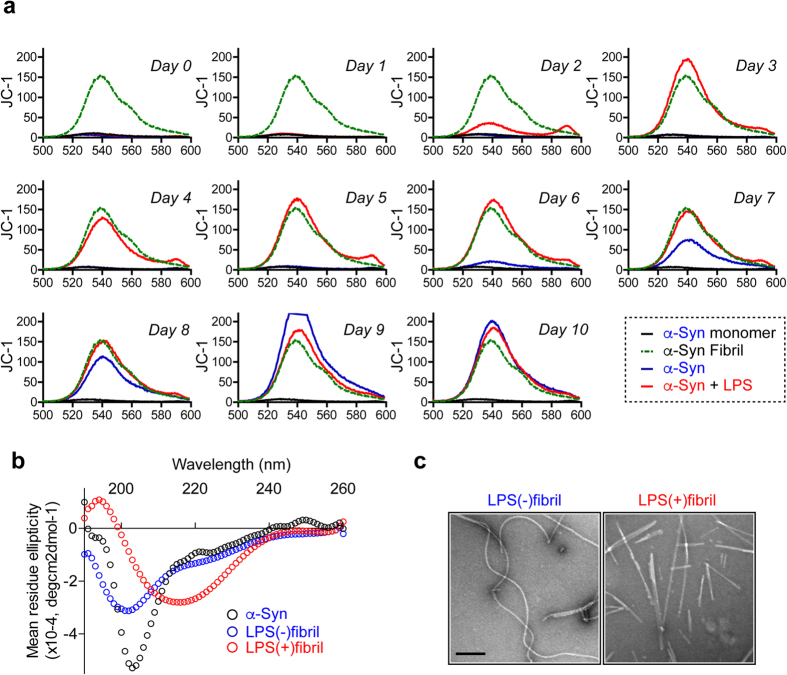
Structural analysis of two distinct α-synuclein fibril polymorphs. Endotoxin-free recombinant human α-synuclein was incubated in the presence or absence of LPS for 10 days. (**a**) Daily JC-1 fluorescence analysis of LPS(−) and LPS(+)α-synuclein. α-synuclein fibril, made of recombinant α-synuclein without endotoxin-removal and endotoxin-free α-synuclein monomer were used as controls. (**b**) Circular dichroic spectra of 9-day old LPS(−) and LPS(+)fibrils. The spectra of monomeric α-synuclein indicates a highly random coil enriched conformation, and the spectrum of LPS(+)fibril indicates a highly β-sheet enriched conformation. The spectra of LPS(−)fibril displayed an intermediate spectrum, indicating less β-sheet and more random coil than in the LPS(+)fibril. (**c**) Electron microscopy analysis of 9-day LPS(−) and LPS(+)fibrils. Scale bar, 200 nm.

To confirm the aggregation kinetics, we assayed fibrillation with another fibril-binding dye, JC-1, which has been shown to selectively bind to fibrils and emit fluorescence at 540 nm^17^. JC-1 binding assay revealed that both LPS(−) and LPS(+)α-synuclein generated JC-1-positive fibrils over time with the former exhibiting slower kinetics (Fig. 2a). The extents of increase of JC-1 fluorescence correlated well with those of the monomer consumption for both LPS(−) and LPS(+)α-synuclein.

Circular dichroism (CD) analysis exhibited different spectra with 9-day-old LPS(−) and LPS(+)α-synuclein (Fig. 2b), with the latter exhibiting a stronger signature for β-sheet than the former (Fig. 2b). This indicates differences in the secondary structures during the aggregation in the presence and absence of LPS. Electron microscopy (EM) revealed filamentous structures for both LPS(−) and LPS(+)α-synuclein (Fig. 2c); however, these fibrils exhibited apparent morphological differences at the ultrastructure level. LPS(−)α-synuclein were curvy and flexible, whereas LPS(+)α-synuclein were ribbon-like with flat and straight morphology (Fig. 2c). These α-synuclein fibrils are similar in morphology to the previously reported α-synuclein fibril strains, e.g., LPS(−)α-synuclein similar to the “fibrils” and LPS(+)α-synuclein to the “ribbons”^18^.

Collectively, these results indicate that in the presence or absence of LPS, α-synuclein produced two different types of fibrils. In the absence of LPS, α-synuclein produced flexible fibrils (LPS(−)fibril) with weak ThT binding, while in the presence of LPS, α-synuclein produced ribbon-like fibrils (LPS(+)fibril) with higher β-sheet contents and strong ThT binding.

### *In vitro* replication of fibril polymorphs

**Figure 3:**
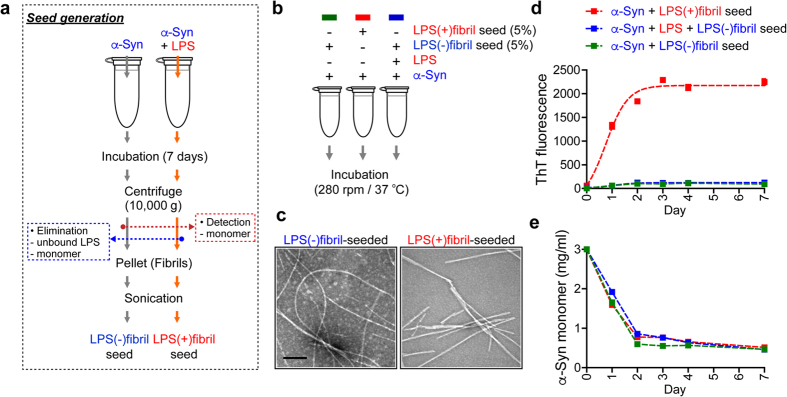
Self-propagation of two distinct α-synuclein fibril polymorphs. (**a**) Preparation of LPS(−) and LPS(+)fibril seeds. Endotoxin-free α-synuclein was incubated in the absence or presence of LPS (100 μg/ml). After a 7-day incubation, each fibril (pellet) was isolated by centrifugation. Soluble α-synuclein monomer and unbound LPS were discarded using wash/centrifugation method. Fibrils were briefly sonicated before use. (**b**) Scheme of seeding experiment. Endotoxin-free recombinant α-synuclein was incubated with LPS(−)fibril seeds (5%) or LPS(+)fibril seeds (5%) in the presence or absence of LPS for 1 week. Data are representative of three independent experiments. (**c**) Electron microscopy was performed with 6-day samples. Scale bar, 200 nm. (**d,e**) ThT fluorescence analysis (**d**) and monomer consumption (**e**) were performed with daily samples.

To determine whether these two types of fibril polymorphs can propagate their respective conformations, we performed seeding experiments. To generate different types of α-synuclein fibril seeds, endotoxin-free α-synuclein was incubated with or without LPS (Fig. 3a). After a 7-day incubation, fibrils were isolated by centrifugation and non-bound free LPS was removed from the fibril preparations by repeated centrifugation/wash cycles (Fig. 3a). The amounts of fibrils were calculated based on the amounts of α-synuclein remaining in the supernatant (Supplementary Fig. 2a). The LPS(−) and LPS(+)fibrils were sonicated briefly before the seeding experiment. The levels of ThT fluorescence were not changed by sonication (Supplementary Fig. 2b). Each seed fibril was incubated with endotoxin-free α-synuclein monomers in the presence or absence of LPS, and the fibrillation was monitored with ThT fluorescence, monomer consumption, and EM (Fig. 3b–e, Supplementary Fig. 3a–c). When α-synuclein monomers were seeded with LPS(+)fibril, they produced LPS(+)fibril-type fibrils, that is ThT-positive, ribbon-like fibrils, even without LPS (Fig. 3c,d). When the aggregation was seeded with LPS(−)fibril, the resulting fibrils exhibited the characteristics of LPS(−)fibrils, flexible fibrils with weak ThT binding (Fig. 3c,d, Supplementary Fig. 3b,c). LPS(−)fibril seeds produced the LPS(−)fibril-type fibrils even in the presence of LPS (Fig. 3b–d), demonstrating that conformations of seeds were faithfully replicated. Monomer consumption was almost identical among the above incubations (Fig. 3e, Supplementary Fig. 3c), indicating the kinetics of seeded aggregation are identical.

Fibril polymorphs, including infectious prion strains, are often characterized by distinct fragmentation patterns upon partial protease digestion. We performed limited protease digestion with LPS(−) and LPS(+)fibrils (Supplementary Fig. 3d). These fibrils were incubated with different concentrations of proteinase K and trypsin, and the resulting fragments were analyzed by sodium dodecyl sulfate-PAGE analysis. With proteinase K digestion, LPS(+)fibril generated at least one extra fragment that was not found in LPS(−)fibril (Supplementary Fig. 3d, left panel, black arrowhead). On the other hand, trypsin treatment generated several extra fragments from both the LPS(+)fibril (black arrowheads) and LPS(−)fibril (white arrowheads), respectively (Supplementary Fig. 3d, right panel). These results suggest that LPS(−) and LPS(+)fibrils are polymorphs with distinct conformations that can be faithfully self-propagating their respective structures.

### Atomic level demonstration of structural differences between the fibril polymorphs

**Figure 4:**
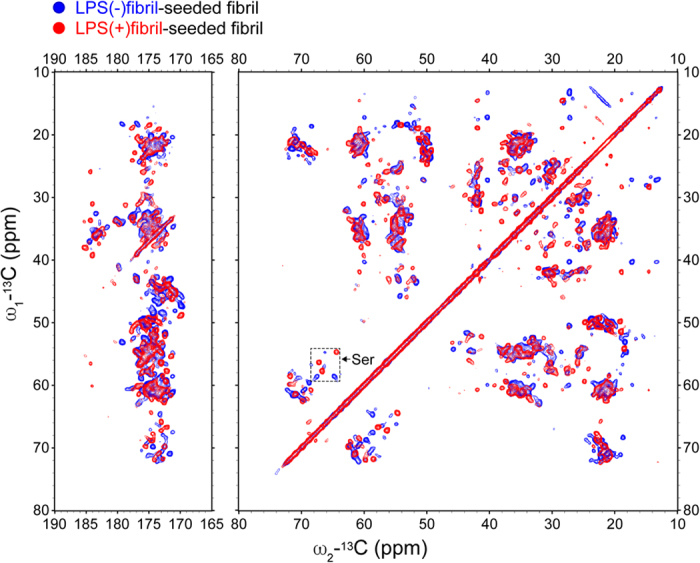
Comparison of 2D 20 ms mixing time DARR spectra of uniformly ^13^C/^15^N-labeled α-synuclein fibrils generated by seeding with two types of fibrils. The two types of fibrils, LPS(−)fibril (blue) and LPS(+)fibril (red) were obtained under identical fibrillation conditions. Significant differences are observed throughout the spectra, and are especially clear in the serine region (cross peaks between ~54–59 PPM and ~64–69 PPM, marked by the hashed box). The left panel displays cross peaks between the aliphatic and carbonyl regions, while the right panel displays cross peaks within the aliphatic region.

In order to compare the structures of these fibril polymorphs at the atomic levels, we performed solid state nuclear magnetic resonance (SS-NMR) spectroscopy. Fibrils generated by seeding monomeric solutions of uniformly ^13^C/^15^N-labeled α-synuclein using LPS(−) or LPS(+)fibril seeds gave rise to different 2D SSNMR DARR spectra (Fig. 4). Intriguingly, the LPS(+)fibril spectra were identical to those previously reported^18^ for a fibril form in which the N-terminal ~40 residues of the protein are incorporated into the fibril core in the form of β-sheet rich structure, whereas the LPS(−)fibril spectra were highly similar to a different previously reported fibril form^19^ in which the fibril core does not include the N-terminal ~40 residues (Supplementary Fig. 4a,b). These observations confirm that the LPS(−) and LPS(+)fibrils feature distinct molecular structures, and are consistent with the ThT and CD data above in that the LPS(−)fibril incorporate less of the protein into the β-sheet rich fibril core, and therefore feature a lower degree of overall β-sheet structure.

### Molecular basis for influence of LPS on fibril morphology

In order to determine how LPS could interact with α-synuclein to influence fibril morphology, we acquired solution state NMR proton-nitrogen correlation spectra of monomeric α-synuclein in solution in the presence of 0, 0.1, 0.5, or 2.5 mg/ml LPS. 0.1 mg/ml of LPS, the same concentration used in the fibrillation reactions, did not lead to any detectable changes in the solution state NMR spectrum, indicating that at this concentration, the interaction of LPS with the protein is too weak/transient to be detected (Supplementary Fig. 5a). At higher concentrations (0.5 or 2.5 mg/ml) the presence of LPS lead to the disappearance of a large fraction of the signals in the NMR spectrum of monomeric α-synuclein (Supplementary Fig. 5b). The remaining resonances all originate from the highly charged C-terminal tail of α-synuclein (Supplementary Fig. 5c), indicating that LPS interacts with the N-terminal membrane-binding domain of α-synuclein, which is constituted by the first ~100 residues of the protein.

### Internalization and degradation kinetics of fibril polymorphs in neuronal and microglial cells

**Figure 5:**
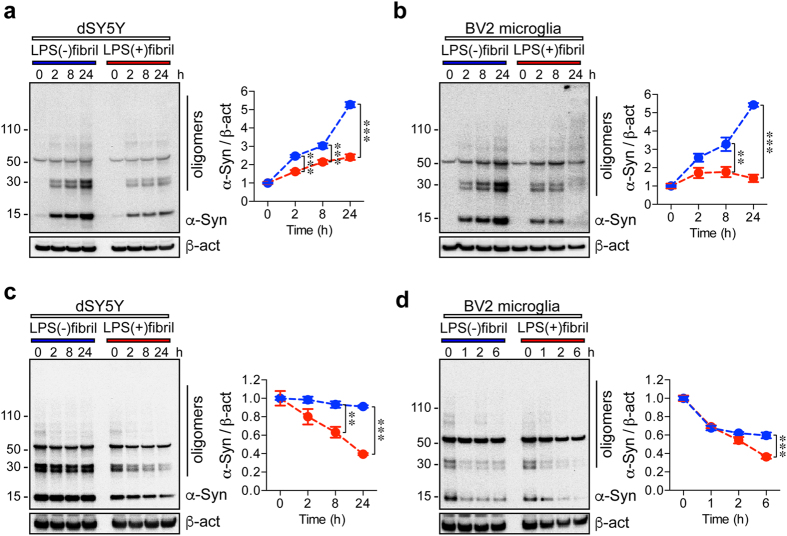
Uptake and degradation kinetics of two different α-synuclein strains by neuronal and microglial cells. (**a,b**) dSY5Y (**a**) and BV2 microglial (**b**) cells were treated with LPS(−) and LPS(+)fibrils for indicated hours. The internalized α-synuclein was determined by western blot analysis (n = 3). (**c,d**) Degradation of internalized α-synuclein in dSY5Y and BV2 cells were determined by western blot analysis (n = 3). dSY5Y (**c**) and BV2 (**d**) cells were treated with either LPS(−) or LPS(+)fibrils for 24 or 2 hours, respectively. After PBS wash, whole cell lysates were harvested at indicated time points, and were analyzed by western blot analysis (n = 3). Data show mean ± s.e.m., ***p < 0.001 and **p < 0.01, one-way ANOVA.

To verify the internalization and degradation kinetics of fibril polymorphs in the cells, we performed uptake and degradation assay using neuronal differentiated SH-SY5Y (dSY5Y) and BV2 microglia cells (Fig. 5). For uptake assay, dSY5Y and BV2 cells were treated with either LPS(−) or LPS(+)fibrils (200 nM) for indicated time (Supplementary Fig. 6a,b, upper panel). The whole cell lysates harvested at various time points were analyzed by western blot analysis to detect internalized α-synuclein (Fig. 5a,b). In dSY5Y cells, the internalization kinetics of both LPS(−) and LPS(+)fibrils showed similar pattern, increased in proportion to exposure time (Fig. 5a). However, the amounts of internalized α-synuclein in LPS(−)fibril treated dSY5Y are significantly greater than the one from LPS(+)fibril treated cells (Fig. 5a). In BV2 cells, LPS(−)fibril showed similar internalization kinetics pattern with dSY5Y cells (Fig. 5b). However, the internalization kinetics of LPS(+)fibril did not show time-dependent increase (Fig. 5b). Moreover, the levels of internalized α-synuclein were clearly decreased at 24-hour time point in LPS(+)fibril treated BV2 cells (Fig. 5b).

To compare the cellular degradation kinetics of LPS(−) and LPS(+)fibrils, dSY5Y and BV2 cells were treated with each fibrils for 24 and 2 hours, respectively (Supplementary Fig. 6a,b, lower panel). After elimination of fibrils, whole cell lysates were harvested at the indicated time points, and were analyzed by western blot analysis (Fig. 5c,d). Surprisingly, the amounts of internalized LPS(−)fibril was not decreased in dSY5Y for 24 hour, while the amounts of internalized LPS(+)fibril were decreased in inverse proportion to incubation time (Fig. 5c). In BV2 cells, the amounts of internalized LPS(−) and LPS(+)fibrils were decreased in inverse portions to incubation time (Fig. 5d). However, small portion of internalized LPS(−)fibrils were still remained at 6-hour time point in the BV2 cells, while most of internalized LPS(+)fibrils had disappeared at the time point (Fig. 5d). Taken together, these results support that two distinct fibril polymorphs have different neuronal and microglial internalization/degradation kinetics.

### Fibril polymorphs generate distinct pathological phenotypes in mice

**Figure 6:**
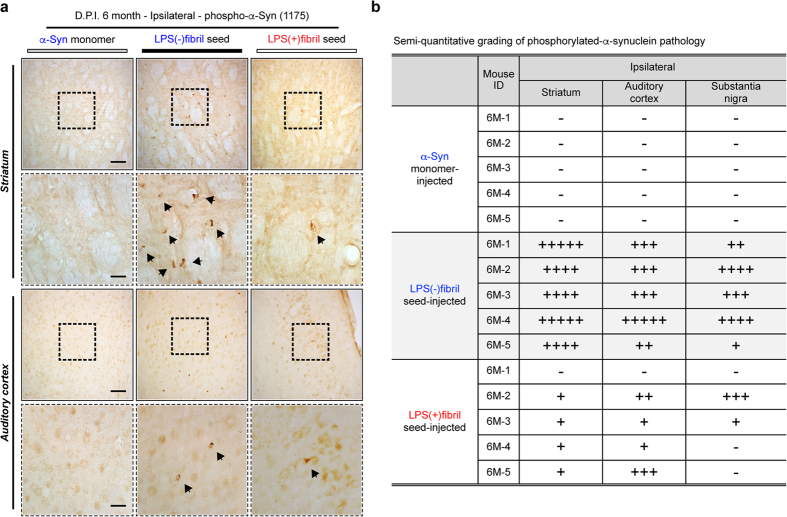
Generation of distinct α-synucleinopathies by two different α-synuclein strains. (**a,b**) Wild type mice were injected with α-synuclein monomer, LPS(−)fibril seed or LPS(+)fibril seed, and distributions of phosphorylated-α-synuclein were analyzed by immunohistochemical analysis at 6 months after injection. Sections were immunolabeled with anti-phospho-α-synuclein (1175) antibody (n = 5 per each group). (**a**) Representative low-power (upper panels) and high-power (lower panels) images of ipsilateral striatum and auditory cortex. Arrows highlight phospho-α-synuclein-positive structures. Scale bars, 50 μm (low-power) and 25 μm (high-power). (**b**) Semi-quantitative grading of α-synuclein pathology in the injected mice.

Since we have two distinct, sustainable polymorphs which have strikingly different cellular internalization/degradation kinetics with structural information in sufficient details, we injected LPS(−) and LPS(+)fibrils as seeds into the striatum of naïve mice (C57BL/6J) and analyzed pathological phenotypes at 3- and 6-month post-injection. Inoculation of these seeds induced widespread distribution of phosphorylated-α-synuclein (pαSyn) pathology throughout the brains, including striatum, substantia nigra, amygdala, and auditory cortex, while injection of α-synuclein monomer failed to generate these changes (Fig. 6a,b, Supplementary Fig. 7). Distribution of pαSyn structures became wider as the duration post injection increased; however, it did not resulted in striatal neurodegeneration (Supplementary Fig. 8a–c). Remarkably, quantitative analysis revealed that these seeds induced different patterns of pathological distribution and severity (Fig. 6b). LPS(−)fibril seeds induced a widespread and strong pαSyn pathology throughout the brain (Fig. 6a,b, Supplementary Fig. 7). In contrast, LPS(+)fibril seeds induced widespread but a weaker pαSyn pathology than its counterparts (Fig. 6a,b, Supplementary Fig. 7). LPS(+)fibril seeds induced relatively strong accumulation of α-synuclein in the striatum and auditory cortex, while LPS(−)fibril affected various regions evenly and more strongly (Fig. 6b).

**Figure 7:**
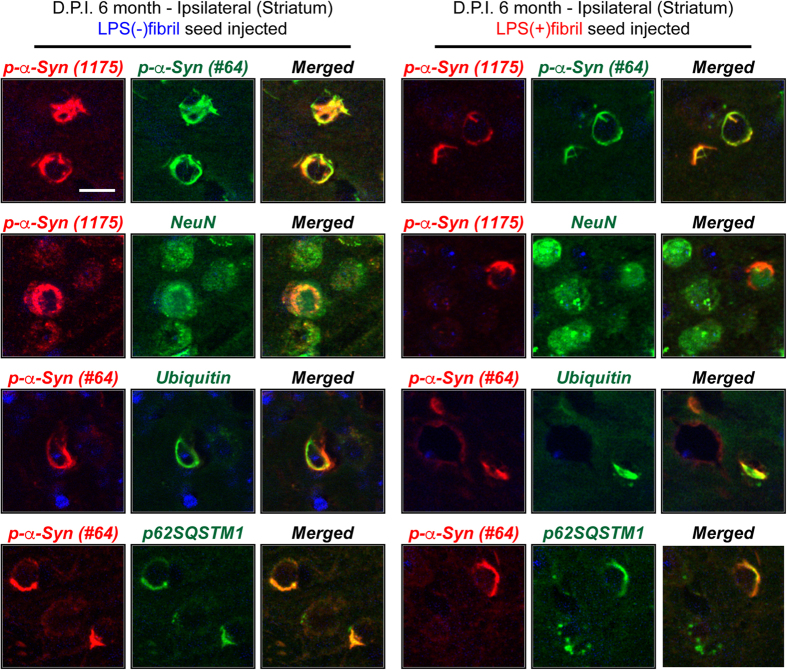
Characterization of the phosphorylated-α-synuclein structures in seeds-injected mice brain. The phosphorylated-α-synuclein structures in the striatum of seed-injected mice were analyzed by double-immunolabeling analysis. The brain sections were incubated with one of following primary antibodies combinations; p-α-Syn (1175)/p-α-Syn (clone#64), p-α-Syn (1175)/NeuN, p-α-Syn (clone#64)/ubiquitin, and p-α-Syn (clone#64)/p62SQSTM1. Scale bar, 25 μm.

**Figure 8:**
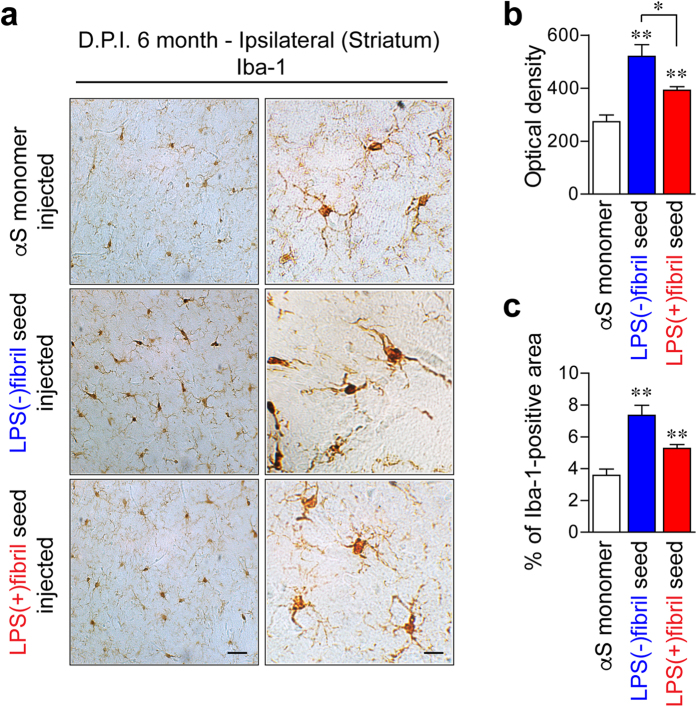
Microglial activation in seed-injected mice brain. Microglia activation were analyzed by immunohistochemical analysis 6 months after injection. Immunoreactivity against Iba-1 was analyzed in ipsilateral striatum of the brains (n = 5 per group). (**a**) Representative images of ipsilateral striatum. (**b,c**) Optical density of Iba-1 immunoreactivity (**b**) and percentage of Iba-1-positive area (**c**). Data show mean ± s.e.m., **p < 0.01 and *p < 0.05, one-way ANOVA, scale bar, 25 μm.

To further characterize the pαSyn-positive structures, the brain sections from seeds-injected mice were analyzed by double-immunolabeling analysis (Fig. 7). In order to avoid the cross-reactivity of the anti-pαSyn antibody (Clone #81A) with other cellular components^20^, we used two different anti-pαSyn antibodies. Clone (1175)^21^ was generated by co-authors of this report and the other one was purchased from Wako Pure Chemical industries, Ltd. (Clone #64). The pαSyn structures were completely co-labeled with both anti-pαSyn antibodies (Fig. 7, first row panels). In addition, double-immunolabeling analysis revealed that the structures were co-localized with NeuN (Fig. 7, second row panels), ubiquitin (Fig. 7, third row panels), and p62SQSTM1 (Fig. 7, fourth row panels), but not with tau (Data not shown). Injections of both fibril seeds also increased microglial activation, with LPS(−)fibril seeds inducing stronger glial activation than LPS(+)fibril seeds (Fig. 8a–c). Similar to *in vivo* results, treatment of LPS(+)fibril induced strong activation of BV2 microglial cells, resulted in overexpression of cytokine genes, such as IL-1β, TNFα, and IL-6 (Supplementary Fig. 9a–c). However, although inoculation of LPS(−)fibril seed induced mild microglia activation in the mice, treatment of LPS(−)fibril did not alter the expression of cytokines in BV2 cells (Supplementary Fig. 9a–c). Collectively, these results show that natural pathogens, such as bacterial endotoxins, generate fibril polymorphs that are structurally and characteristically distinct at the atomic levels. Importantly, these polymorphs induced distinct pathological phenotypes *in vivo* upon intracerebral injection, establishing the endotoxin-induced fibril polymorphs as a pathogenic strain.

## Discussion

Previous studies have demonstrated various types of α-synuclein fibrils with distinct conformational characteristics^7^,^22^. Conformational propagation of these fibril types has been demonstrated in test tubes by showing conversion of recombinant monomers to the respective seed fibrils. Amyloid fibrils induce proteinopathies in animal brains upon injection^21^,^23^, and studies on prions have shown that infections prions with distinct structures (strains) caused distinct pathological changes in animals and stably propagate the pathogenic characteristics horizontally. α-synuclein fibrils have also been shown to induce synucleinopathy lesions upon injection into mouse brains^21^,^23^. One of these studies described strains of α-synuclein fibrils^24^; however, the specific link between structures and pathological phenotypes has not been addressed. The most significant contribution of our study is the demonstration of the relationship between fibril conformations and pathological phenotypes. We showed that α-synuclein fibrils with distinct structures at the atomic levels induced distinct pathological phenotypes *in vivo* upon intracerebral injection. The relationship between specific fibril conformations and pathological phenotypes established these distinct α-synuclein fibrils as pathogenic strains. Recently, Peelaerts *et al*. reported that α-synuclein fibril subtypes, “fibrils” and “ribbons”, exhibited different neurotoxic properties and induced different patterns of aggregate deposition^9^. Importantly, we demonstrated that natural pathogens, such as bacterial endotoxins, could generate such strains. These results might provide an explanation as to how environmental exposures can generate different types of pathology, hence the clinico-pathological disease heterogeneity.

Strain characteristics have been used as an argument for seeded protein aggregation as the mechanism of aggregate spreading^25^; however, other possibilities remain plausible as well. Aggregate strains may induce specific pathogenic microenvironments in particular brain regions and cell types in which specific patterns of synucleinopathy occur. Structural characteristics of aggregates may be propagated by adopting conformations that are compatible within the microenvironments. In this regard, inflammation may be a mediator for creating such microenvironments. However, the mechanism underlying the link between structure and pathology in a particular strain remains to be elucidated.

The NMR spectrum in the presence of 2.5 mg/ml LPS is nearly identical to that observed in the presence of lipid vesicles or detergent micelles at the same temperature^26^, suggesting that the α-synuclein may be binding to LPS micelles, which are known to form in these concentration ranges^27^. Although it is difficult to gain any conclusive insights from these results into the mechanisms by which LPS can influence fibril morphology, there is evidence micelle- and membrane-bound forms of α-synuclein facilitate its aggregation^28^,^29^ and this enhancement depends on structures formed at the very N-terminus of the protein^30^. This could potentially explain why the LPS(+)fibril structures incorporate the ~40 N-terminal residues of the protein in the fibril core.

Our LPS(−)fibril shows lower β-sheet content than the LPS(+)fibril, and when injected into the striatum of mice, LPS(−)fibril spread much more robustly and induced inflammatory responses more strongly than the LPS(+)fibril. These *in vivo* properties of LPS(−) and LPS(+)fibrils are interesting for two reasons. A recent report by Chen *et al*. showed a stable amyloid oligomer of α-synuclein with less β-sheet content than fibrils was more toxic and damaging to membranes than fibrils^31^. This study and ours seems to suggest that lower β-sheet content, hence more flexibility in structure, is associated with higher pathogenicity. Secondly, Kubota *et al*. showed that prion transmission was more efficient with less β-sheet structure, resulting in more fragile fibrils generating more nuclei^32^. Consistent with the conclusion of this prion study, the more robust and wider spreading pathology shown in animals injected with LPS(−)fibril than in those with LPS(+)fibril may suggest that fibrils with less rigid structure serve as better pathological agents.

Staging of Lewy pathology proposed by Braak and colleagues implicated the role of the gastrointestinal (GI) system and olfactory bulb (OB) in the initiation of α-synuclein pathology^33^. Neurons in the enteric nervous system (ENS) of the GI tract or the chemosensory receptor neurons in the OB may be exposed directly to the external environment, including bacterial endotoxins or other environmental toxins. One could speculate that neurons in the ENS and OB generate distinct strains of fibrils depending on the types of environmental exposure. These strains could be transported to the CNS and cause distinct types of pathology. Our results support the concept that environmental exposures may generate unique α-synuclein fibril strains, which then spread to the CNS leading to distinct pathological phenotypes and perhaps to different α-synuclein-related diseases.

## Materials and Methods

### Preparation of endotoxin-free recombinant α-synuclein

Preparation of human recombinant α-synuclein has been described elsewhere^34^. Bacterial endotoxins were removed using the Toxineraser endotoxin removal kit (GenScript, Piscata way, NJ) according to manufacturer’s instruction^16^. The level of endotoxin was determined using ToxinSensor^TM^ Chromogenic LAL Endotoxin Assay Kit (GenScript). Recombinant α-synuclein contains 10.6 ~ 11.3 endotoxin units per 1 mg of protein and endotoxin-free α-synuclein contains less than 0.015 endotoxin units per 1 mg of protein. The buffer of purified preparation was exchanged to distilled water using SnakeSkin^TM^ Dialysis tubing system (10K, Life Technologies, Grand Island, NY) according to manufacturer’s instruction at 4 °C. Following buffer exchange, preparations were lyophilized and stored at −80 °C until use.

### Thioflavin T fluorescence assay

Thioflavin T fluorescence was measured using SpectraMax GEMINI EM (Molecular devices, Sunnyvale, CA) as described elsewhere^34^. Briefly, 10 μM of protein samples were incubated with 10 μM of thioflavin T. After a five-minute incubation, the fluorescence was measured at an excitation of 450 nm and emission at 490 nm.

### JC-1 fluorescence assay

To monitor the α-synuclein fibrillation states (monomer, oligomeric intermediate, and fibril forms), we performed JC-1-binding fluorescence assay using luminescence spectrophotometer (LS-55; Perkin-Elmer, Waltham, MA)^17^. Briefly, 10 μl of 10 μM JC-1 in 100% DMSO and 20 μl of 50 μM α-synuclein fibrils were mixed with 170 μl of 20 mM MES buffer at pH 6.5. The JC-1- binding fluorescence spectrum was measured between 500 and 600 nm with excitation at 490 nm. The slit width for both excitation and emission was 5 nm. Each of the spectrum recorded was an average of three separate scans measured with the scan speed of 100 nm/min.

### Transmission electron microscope (TEM)

α-synuclein fibrils were observed with TEM (JEM-1010; JEOL, Tokyo, Japan). First, 20 μl of the sample was adsorbed onto a carbon-coated copper grid (200 mesh) then air dried for 5 minutes. To visualize the amyloid fibril sample on TEM, the grid was negatively stained with 2% uranyl acetate in a darkroom.

### Circular dichroism (CD) analysis

The secondary structures of LPS(−) and LPS(+) α-synuclein were evaluated using Chirascan-plus CD spectrometer (Applied Photophysics Ltd, Leatherhead, UK). α-synuclein monomer and fibril were used as negative and positive controls, respectively. Before analysis, samples were diluted to 7 μM with 20 mM MES buffer at pH 6.5. The spectrum was measured within a 1.0 mm-path length quartz cuvette between 185 and 260 nm with the scan rate of 20 nm per minute and the step resolution of 1.0 nm. The resulting spectrum was an average of five separate scans.

### Generation of LPS(−) and LPS(+)fibrils

To generate LPS(−) and LPS(+)fibrils, lyophilized endotoxin-free recombinant α-synuclein was dissolved in phosphate buffered saline (PBS) and filtered using 0.2 μm pore size syringe filter. The concentration of monomeric α-synuclein was determined by BCA assay. A total of 1 ml of dissolved α-synuclein (200 μM) was incubated in the presence or absence of LPS (100 μg/ml, 055:B5, Sigma-Aldrich, St Louis, MO) at 37 °C with agitation (280 rpm). Fifty microliters were taken from each tube at the same time every day to perform daily analysis, described in Fig.1a. To prepare 7-day LPS(−) and LPS(+)fibril seeds, dissolved α-synuclein was incubated with or without LPS (100 μg/ml) at 37 °C with agitation (280 rpm). After a 7-day incubation, samples were centrifuged at 10,000× g for 10 minutes. Supernatants were transferred to new tubes to calculate the amount of soluble α-synuclein. Pellets were washed with 1 ml of fresh PBS for three times. The total amount of insoluble α-synuclein fibrils were calculated based on the amount of remaining soluble α-synuclein in the supernatant. After the determination of concentration, insoluble α-synuclein fibrils were resuspended with PBS (final concentration; 2 mg/ml) and briefly sonicated.

### Solid state nuclear magnetic resonance (ssNMR)

Uniformly ^13^C/^15^N-labeled α-synuclein was produced as previously described^35^,^36^. Briefly, *Escherichia coli* BL21 (DE3) cells were transformed with a plasmid encoding α-synuclein and grown in rich media at 37 °C to an optical density of ~0.6. Cells were pelleted and resuspended in wash media, and pelleted and resuspended again in minimal media containing ^15^N-labeled ammonium chloride and ^13^C-labeled glucose in the presence of ampicillin (100 μg/ml), and harvested after being induced with isopropyl-β-D-1-thiogalactopyranoside and grown for 3 hours at 37 °C. Proteins were then purified by ammonium sulfate cuts, anion-exchange chromatography, and reversed-phase HPLC. The purified protein was lyophilized and stored at −20 °C.

Lyophilized protein was dissolved in 20 mM MES buffer (pH 6.5) and filtered using a 0.2 μm filter to remove large molecular weight aggregates. Uniformly ^13^C/^15^N-labeled α-synuclein fibrils with LPS(−) and LPS(+)fibril seeds were grown by adding 5% LPS(−) or LPS(+)fibril seeds, respectively. Both fibrils were incubated with a final α-synuclein concentration of 200 μM, in 20 mM MES buffer at pH 6.5, at 37 °C and with continuous shaking at 250 rpm for 9 days.

All ssNMR experiments were conducted using 3.2-mm triple-resonance (^1^H, ^13^C, ^15^N) probe heads on a Bruker AVANCE II 750 MHz/ 17.6T spectrometer at the New York Structural Biology Center (NYSBC). The chemical shifts of ^13^C were calibrated with adamantine as an external reference^37^. All experiments were carried out at a sample temperature of around ^+^278 K and magic-angle spinning speed of 13 kHz. An initial ramped cross-polarization^38^,^39^ was used to transfer the magnetization from ^1^H to ^13^C with a contact time of 900 μs. TPPM high-power proton decoupling^40^ was applied during evolution and detection periods with radiofrequency amplitudes of 86.2 kHz. ^13^C–^13^C magnetization transfer during the mixing time was accomplished using the dipolar assisted rotational resonance (DARR) condition^41^. A mixing time of 20 ms was used to select for intra-residue magnetization transfers. All spectra were processed with Topspin and analyzed in SPARKY version 3.1 (T. D. Goddard and D. G. Kneller, University of California).

### Uptake and degradation assay of α-synuclein fibrils

Differentiated SH-SY5Y (dSY5Y) and BV2 microglia cells were treated with either LPS(−) or LPS(+)fibrils (200 nM) for indicated hours (Supplementary Fig. 6a,b). For uptake assay, whole cell lysates of fibril treated cells were harvested at indicated time points (Supplementary Fig. 6b, upper panel). For degradation assays, cells were pretreated with fibrils for indicated hours, than washed with PBS four times and the subsequently incubated with fresh media. After indicated incubation time points, whole cell lysates were harvested (Supplementary Fig. 6b, lower panel). The cellular internalized α-synuclein fibrils were determined by western blot analysis.

### Stereotaxic injection of α-synuclein seeds and immunohistochemical analysis

Injection of recombinant α-synuclein has been described previously^21^. Briefly, three-month-old female C57BL6/J mice (CLEA Japan Inc., Tokyo, Japan) were anaesthetized with 50 mg/kg pentobarbital sodium before stereotaxic injection. A total of 5 μl of recombinant α-synuclein monomer (10 μg/μl; n = 10), LPS(−)fibril seed (2 μg/μl; n = 10), and LPS(+)fibril seed (2 μg/μl; n = 9) were stereotaxically injected into the striatum (AP; 0.2 mm, ML; −2.0 mm, and DV; −2.6 mm) of the right hemisphere using a 10-μl Hamilton syringe^42^. Three- or six-month post injection, brains were processed for immunohistochemical, double-immunolabeling, and biochemical analysis. All experimental protocols were approved by the Animal Care and Use Committee of the Tokyo Metropolitan Institute of Medical Science (Protocol #13066). All animal experiments were carried out in accordance with the approved guidelines. We found no difference in the severity or distribution of α-synuclein pathology between male and female mice after intracerebral injection (data not shown).

The procedures for immunohistochemical analysis have been described elsewhere^43^. Briefly, blind-coded vibratome brain sections were incubated with the following primary antibodies; polyclonal anti-phosphorylated α-synuclein (1175)^21^, polyclonal anti-Iba-1 (Wako pure chemical industries, Richmond, VA), monoclonal anti-NeuN (Millipore, County Cork, Ireland), and monoclonal anti-tyrosine hydroxylase (TH, Millipore) antibodies. After washing with PBS, sections were incubated with biotinylated-secondary antibodies (Vector Laboratories, Burlingame, CA) and subsequently signal was detected using ABC staining kit (Vector Laboratories). To determine microgliosis and striatal neurodegeneration, the optical density and percentage of Iba-1 positive area, numbers of NeuN-positive cells, and the striatal TH-optical density were analyzed using ImageJ (NIH)^44^.

The procedures for double-immunolabeling analysis have been described elsewhere^43^. Briefly, blind-coded vibratome brain sections were immunolabeled with two of following primary antibodies; polyclonal anti-phosphorylated α-synuclein (1175), monoclonal anti-phosphorylated α-synuclein (Clone #64; Wako pure chemical industries, clone), monoclonal anti-NeuN (Millipore), polyclonal anti-ubiquitin (Dako, Carpinteria, CA), polyclonal anti-p62SQSTM1 (Sigma-Aldrich, St Louis, MO), or polyclonal anti-total-tau (Dako) antibodies. Immunoreactivities were detected with the Tyramide Signal Amplification Direct system (PerkinElmer, Waltham, MA) or FITC-tagged secondary antibodies. Sections were imaged with a Zeiss 63X (N.A. 1.4) objective on an Axiovert 35 microscope (Zeiss, Cambridge, MA) with an attached MRC 1023 LSCm system (Bio-Rad, Hercules, CA).

### Statistical analysis

All statistical analysis was performed using InStat (GraphPad Software, San Diego, CA). All data were analyzed for statistical significance by using one-way ANOVA. All data are represented as means ± s.e.m.

## Additional Information

**How to cite this article**: Kim, C. *et al*. Exposure to bacterial endotoxin generates a distinct strain of α-synuclein fibril. *Sci. Rep.* **6**, 30891; doi: 10.1038/srep30891 (2016).

## Supplementary Material

Supplementary Information
