# Supplementary material for: Exposure to bacterial endotoxin generates a distinct strain of α-synuclein fibril
Source: Sci Rep. 2016 Aug 4;6:30891. doi: 10.1038/srep30891 (PMC4973277; doi:10.1038/srep30891)
Supplement: Supplementary Information [file srep30891-s1.doc]

**Supplementary Information**

**Exposure to bacterial endotoxin generates a distinct strain of α-synuclein fibril**

Changyoun Kim^1,2^, Guohua Lv^3^, Jun Sung Lee^1^, Byung Chul Jung^1,4^, Masami Masuda-Suzukake^5^, Chul-Suk Hong^6^, Elvira Valera^2^, He-Jin Lee^7^, Seung R. Paik^6^, Masato Hasegawa^5^, Eliezer Masliah^2^, David Eliezer^3^ & Seung-Jae Lee^1^

^1^Department of Biomedical Sciences, Neuroscience Research Institute, Seoul National University College of Medicine, Seoul, Korea.

^2^Departments of Neurosciences and Pathology, School of Medicine, University of California, San Diego, La Jolla, CA, USA.

^3^Department of Biochemistry, Weill Cornell Medical College, NY, USA.

^4^Department of Biomedical Laboratory Science, College of Health Science, Yonsei University, Wonju, Korea.

^5^Department of Neuropathology and Cell Biology, Tokyo Metropolitan Institute of Medical Science, Tokyo, Japan.

^6^School of Chemical and Biological Engineering, College of Engineering, Seoul National University, Seoul, Korea.

^7^Department of Anatomy, School of Medicine, Konkuk University, Seoul, Korea.

**
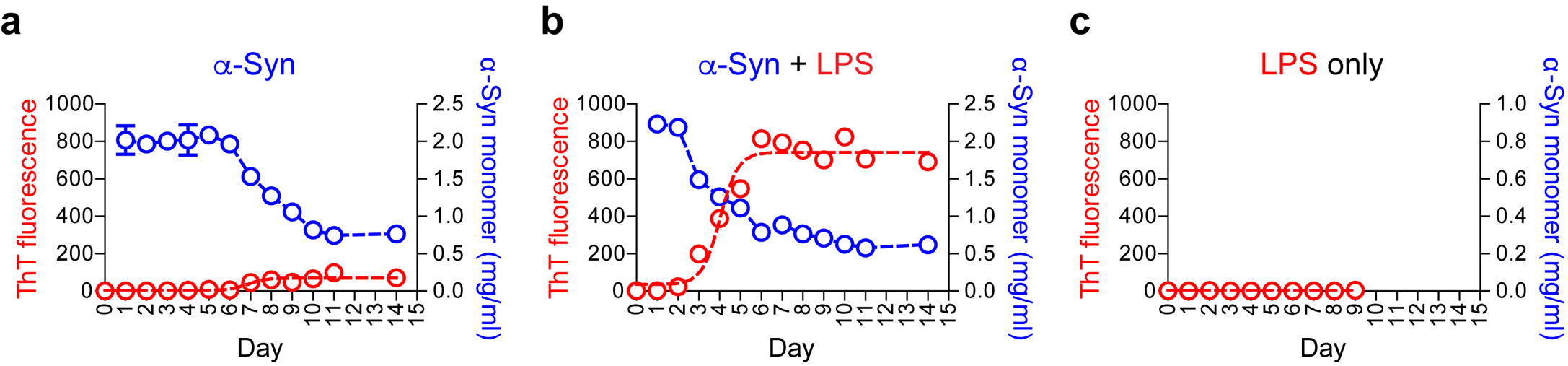
**

**Supplementary Figure 1. Fibrillation kinetics of endotoxin-free α-synuclein in the presence and absence of lipopolysaccharide.** (**a-c**) Endotoxin-free α-synuclein was incubated in the absence (**a**) and presence (**b**) of lipopolysaccharide (LPS, 100 μg/ml) for indicated days. ThT fluorescence reactivity (red) and monomer consumption (blue) were measured in every day. (**c**) LPS was incubated in alone as a control.


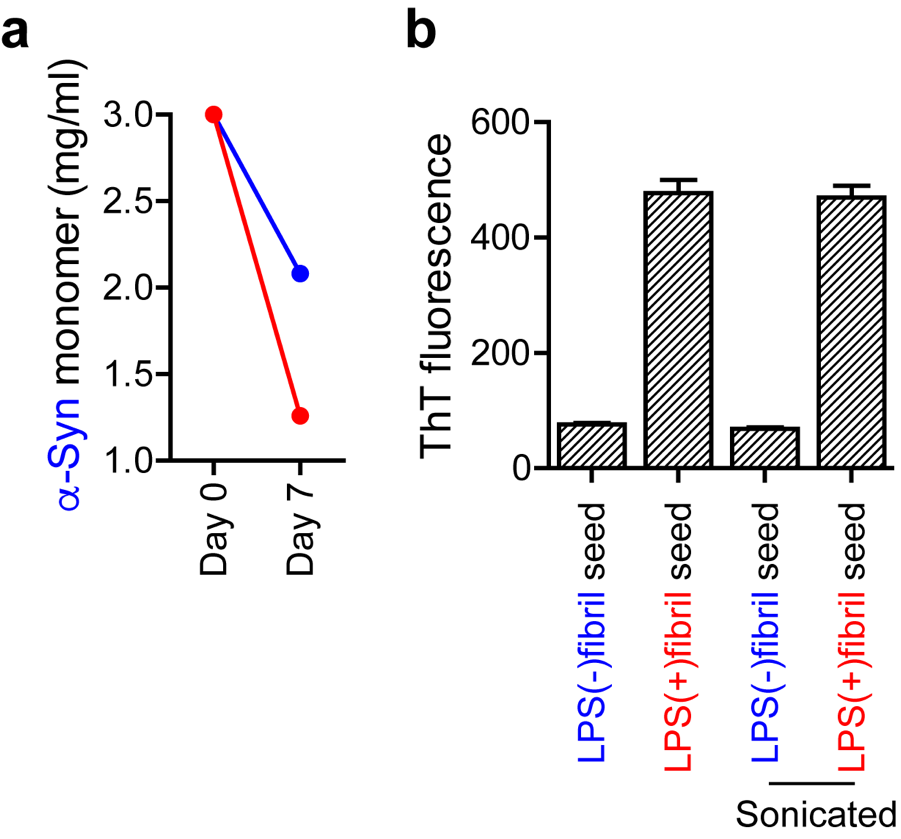


**Supplementary Figure 2. Preparations of LPS(-) and LPS(+)fibril seeds.** Endotoxin-free α-synclein was incubated in the absence and presence of LPS. After a 7-day incubation, each fibrils (pellets) were washed and stored until use. Fibrils were briefly sonicated before use. (**a**) Consumption of soluble α-synuclein monomer. Total amount of fibril was calculated based on the amount of remaining monomers. (**b**) ThT reactivities of fibrils were not affected by sonication.

**
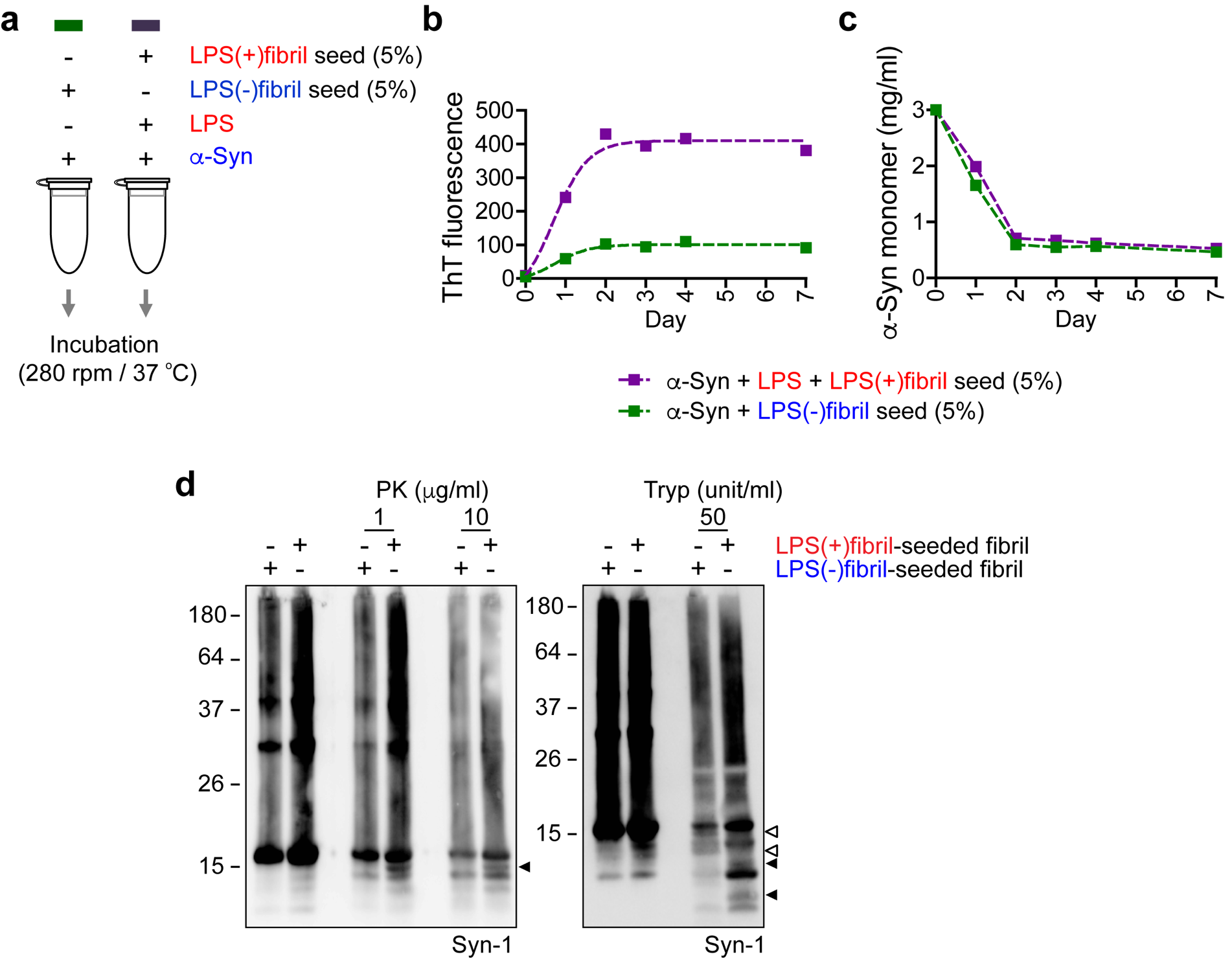
**

**Supplementary Figure 3. Expanded seeding experiment for LPS(-) and LPS(+)fibril seeds.** (**a**) Experimental scheme. Endotoxin-free α-synuclein was incubated with LPS(-)fibril seed (5%) or with LPS(+)fibril seed (5%) in the presence of LPS (100 μg) for 7 days. Daily ThT fluorescence reactivities (**b**) and monomer consumptions (**c**) were determined in indicated days. (**d**) Proteinase K and trypsin resistance analysis of seed-induced fibrils. Extra fragments generated by protease were highlighted with white (LPS(-)fibril) and black (LPS(+)fibril) arrowheads.


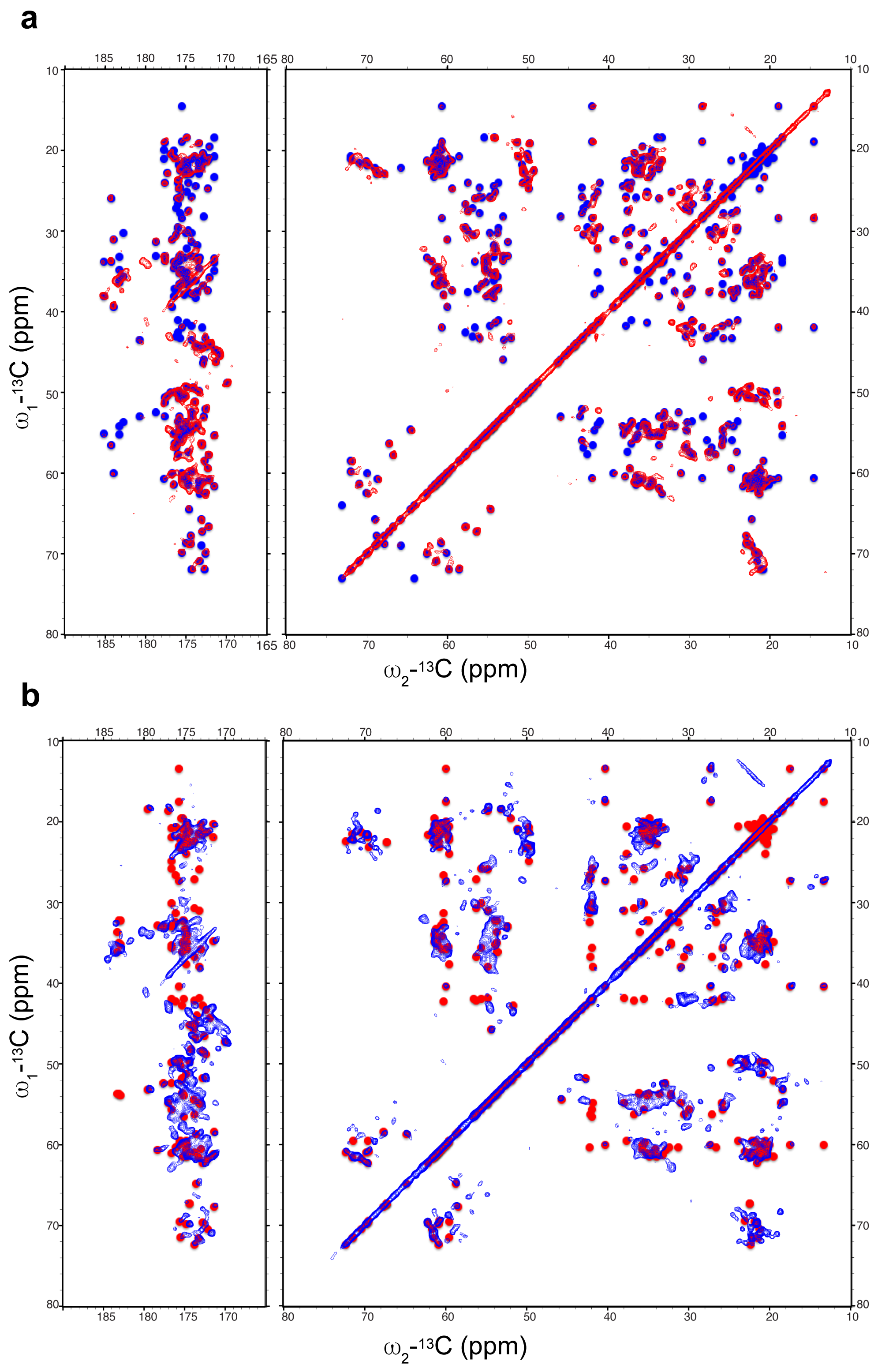


**Supplementary Figure 4. Comparisons of 2D 20 ms mixing time DARR spectra of LPS(-) and LPS(+)fibril seeds-induced fibrils with previously recognized α-synuclein fibril structures.** (**a**) Comparison of 2D 20 ms mixing time DARR spectra of uniformly ^13^C/^15^N-labeled α-synuclein fibrils seeded with LPS(+)fibril seeds (red) and a ^13^C-^13^C correlation spectrum reconstructed from the resonance assignments reported[^1^](#_ENREF_1) for a form of α-synuclein fibrils in which the N-terminal ~40 residues are incorporated into the fibril core (blue). (**b**) Comparison of 2D 20 ms mixing time DARR spectra of uniformly ^13^C/^15^N-labeled α-synuclein fibrils seeded with LPS(-)fibril seeds (blue) and a ^13^C-^13^C correlation spectrum reconstructed from the resonance assignments reported[^2^](#_ENREF_2) for a form of α-synuclein fibrils in which the N-terminal ~40 residues are not incorporated into the fibril core (red).


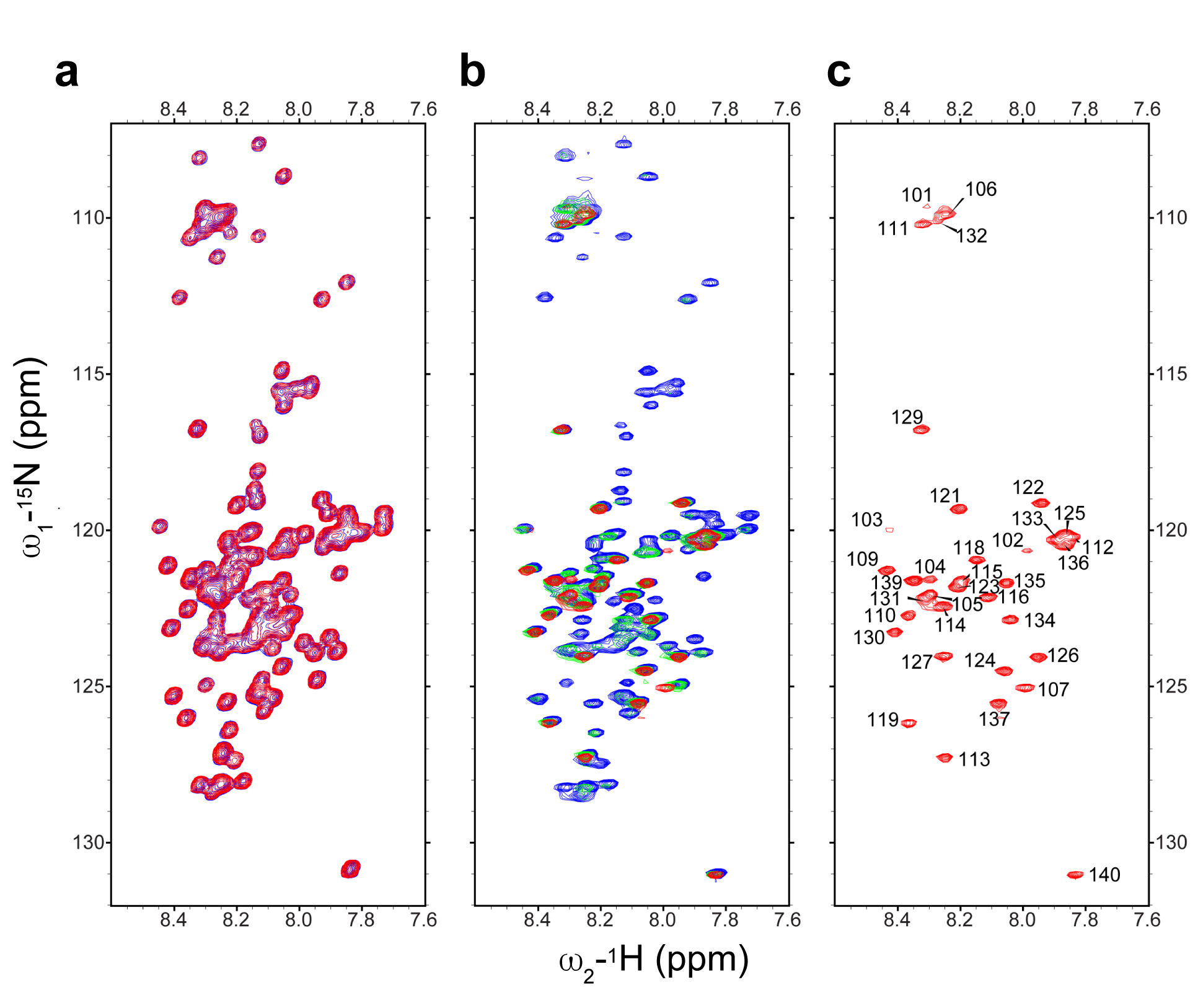


**Supplementary Figure 5. Interactions of LPS with monomeric α-synuclein in solution.** (**a**) NMR proton-nitrogen correlation spectrum of monomeric ^15^N-labeled α-synuclein in the absence (blue) and presence (red) of 0.1 mg/ml LPS. No changes are evident in the spectrum in the presence of LPS. (**b**) NMR proton-nitrogen correlation spectrum of monomeric ^15^N-labeled α-synuclein in the absence (blue) and presence of 0.5 (green) or 2.5 (red) mg/ml LPS. Many of the signals disappear in the presence of these higher LPS concentrations. (**c**) NMR proton-nitrogen correlation spectrum of monomeric ^15^N-labeled α-synuclein in the presence of 2.5 mg/ml LPS annotated with the assignment of each resonance to its position in the α-synuclein amino acid sequence. Only residues from the C-terminal tail of the protein remain visible, while the remainder of the protein is rendered invisible by its interactions with LPS.


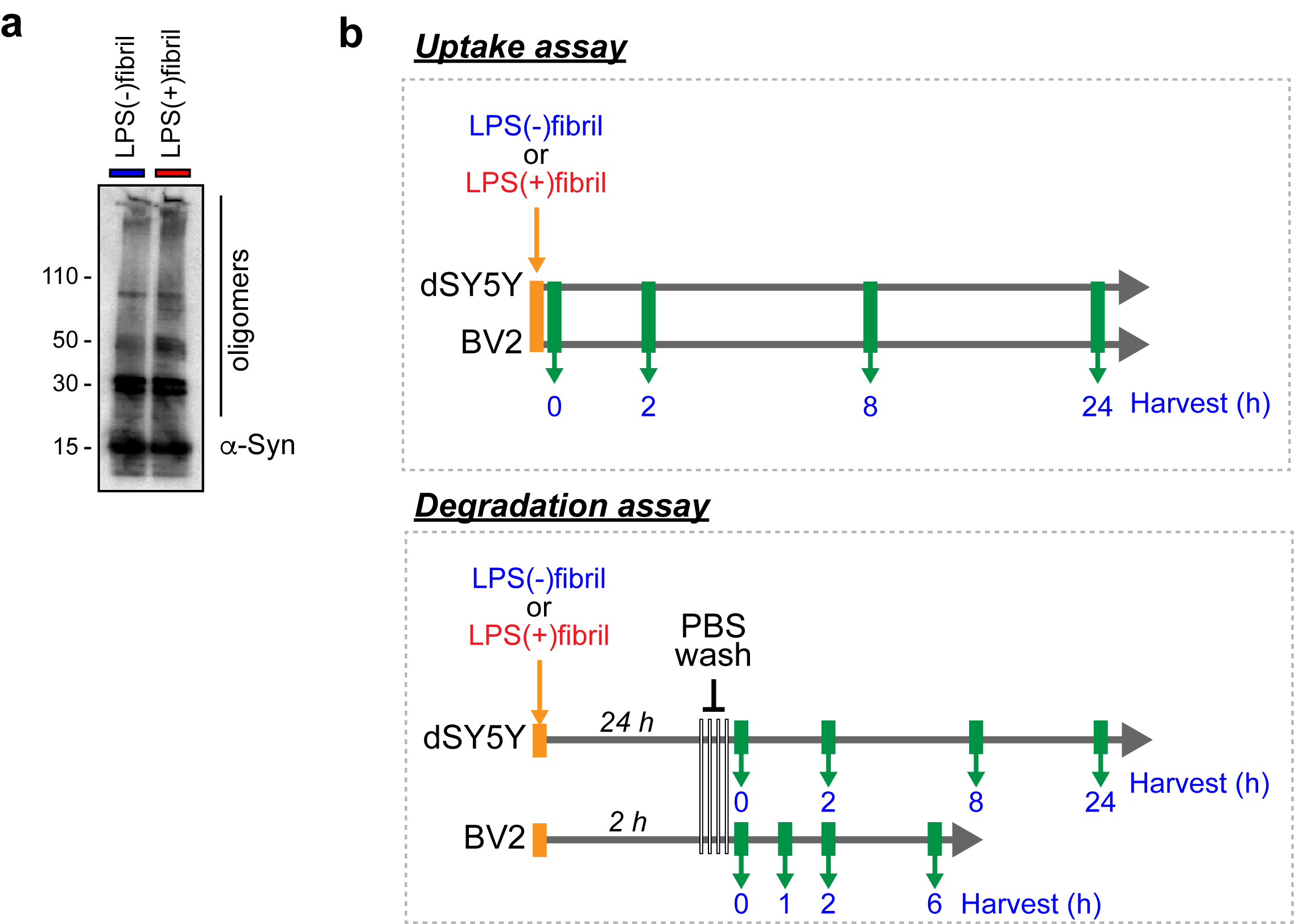


**Supplementary Figure 6. Experimental procedure of α-synuclein fibril uptake and degradation analysis.** (**a**) Western blot analysis of LPS(-) and LPS(+)fibrils. Ten micrograms of fibrils were analyzed using SDS-PAGE gel. (**b**) Experimental schemes of uptake (upper panel) and degradation (lower panel) assays. Differentiated SH-SY5Y (dSY5Y) and BV2 microglia cells were treated with either LPS(-) or LPS(+)fibrils (200 nM). For uptake assay, cells were treated with fibrils, and whole cell lysates were harvested at indicated time points. For degradation assay, cells were pre-treated with fibrils for indicated hours. After a 4-time PBS washing, whole cell lysates were harvested at indicated time points.


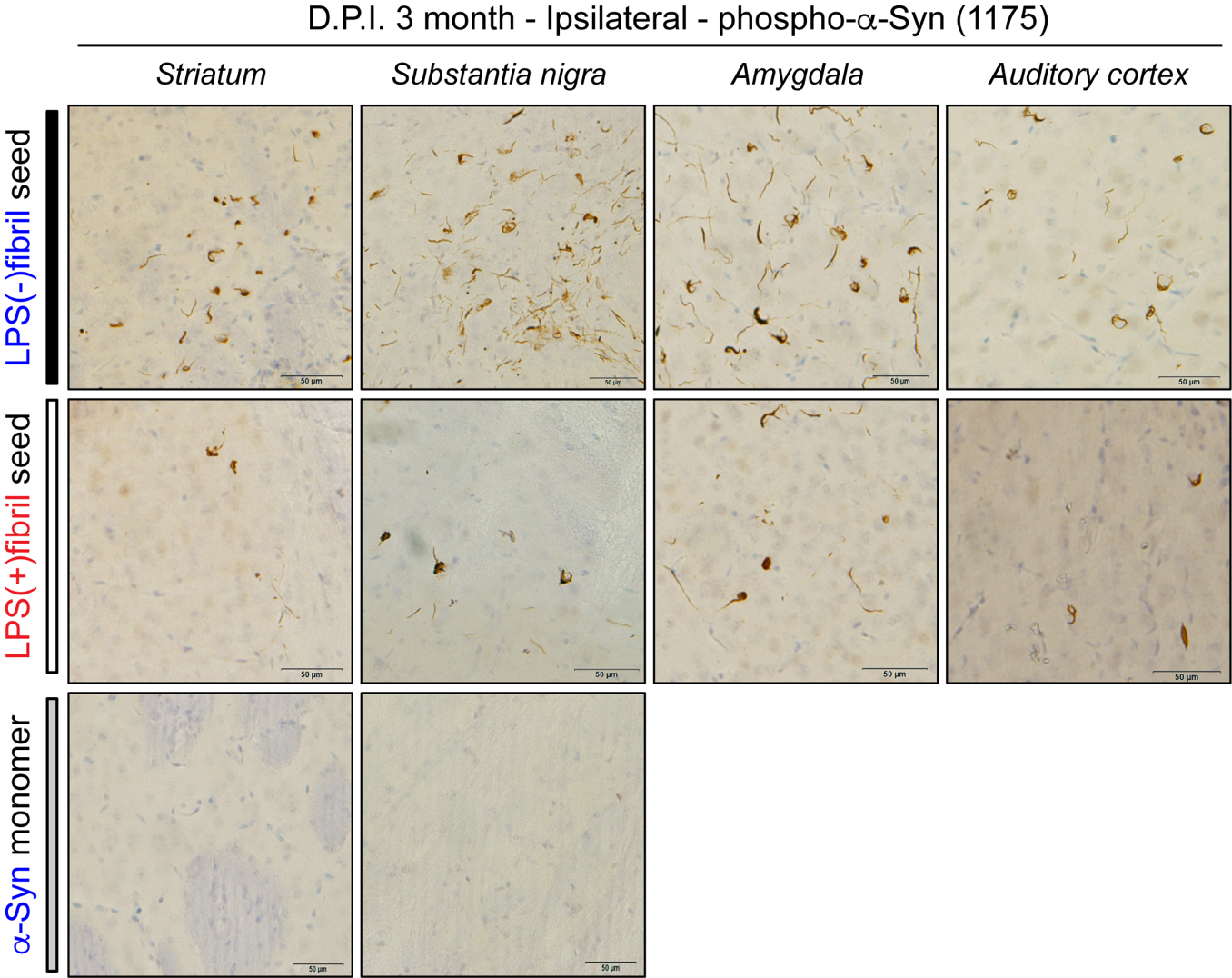


**Supplementary Figure 7. Deposition of phosphorylated-α-synuclein in seeds-delivered mice brains.** Endotoxin-free α-synuclein monomers, LPS(-)fibril seeds, and LPS(+)fibril seeds were injected into striatum of wild type mice brains. Indicated brain regions were immunolabeled with anti-phospho-α-synuclein antibody (1175) after 3 month post injections. Scale bar, 50 μm.


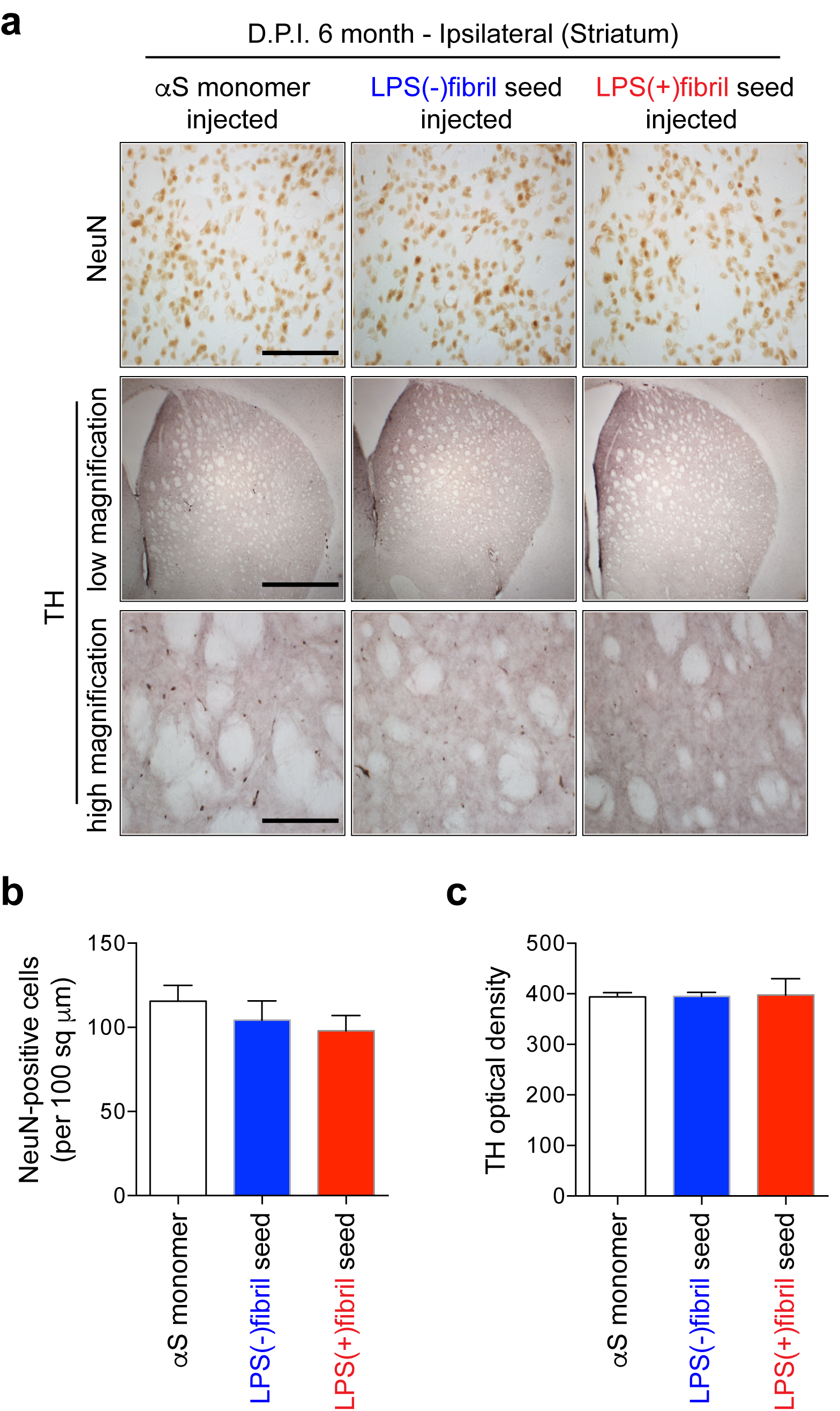


**Supplementary Figure 8. Striatal neuropathology in seed-injected mice.** (**a**) Representative immunohistochemical staining of NeuN (upper panels) and TH (middle and lower panels) in the ipsilateral striatum of seed-injected mice. (**b**) The numbers of NeuN-positive cells in the striatum. (**c**) The levels of dopaminergic fiber in striatum were analyzed by optical density quantification. Data show mean ± s.e.m., n = 5 per each group, scale bars, 25 μm (upper and lower panels) and 250 μm (middle panels).


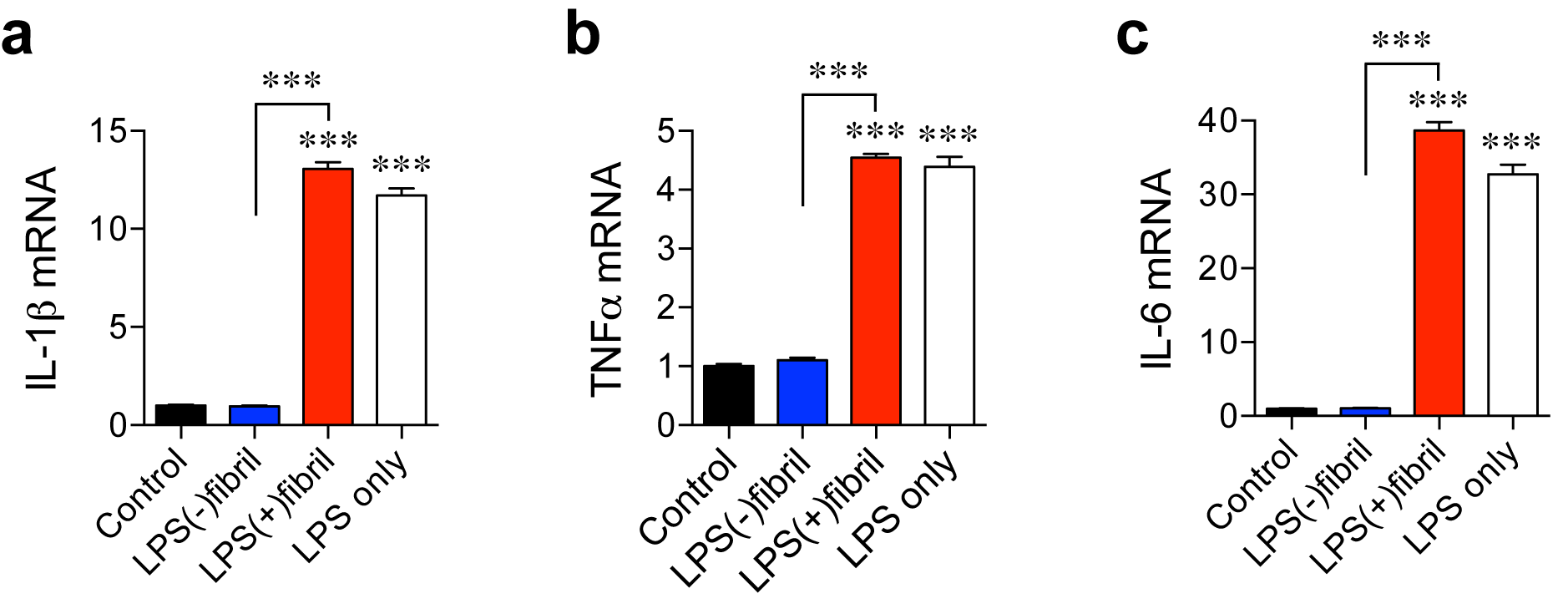


**Supplementary Figure 9. Microglial cytokine genes expressions by LPS(-) and LPS(+)fibrils.** BV2 microglial cells were treated with either PBS (control), LPS(-)fibril (200 nM), LPS(+)fibril (200 nM), or LPS (50 ng/ml) for 6 hours. Total RNA was extracted and reverse-transcribed. The mRNA expression levels of IL-1β (**a**), TNFα (**b**), and IL-6 (**c**) were determined by quantitative realtime PCR analysis. Data show mean ± s.e.m., n = 6 per each group, ***p < 0.001, one-way ANOVA.

**Supplementary Materials and Methods**

**Protease K and trypsin digestions of LPS(-) and LPS(+)fibrils.** Ten micrograms of either LPS(-) or LPS(+)fibrils was incubated with Proteinase K (1, 10 μg/ml) or Trypsin (50 unit/ml) for 10 minutes at 37°C. Proteinase K and trypsin digestions were stopped by addition of PMSF (final concentration; 5 mM) and NuPage LDS sample buffer, respectively. The reaction samples were separated with NuPage 12% Bis-Tris gels (Life Technologies) and transferred to PVDF membrane. To detect α-synuclein, membrane was incubated with monoclonal anti-α-synuclein antibody (Syn-1, BD bioscience, San Jose, CA).

**Solution state nuclear magnetic resonance.** Uniformly ^15^N-labeled α-synuclein was produced as previously described. Briefly, Escherichia coli BL21 (DE3) cells transformed with a plasmid encoding α-synuclein and grown in rich media at 37°C to an optical density of ∼0.6. Cells were pelleted and resuspended in wash media, and pelleted and resuspended again in minimal media containing ^15^N-labeled ammonium chloride and unlabeled glucose in the presence of ampicillin (100 μg/ml), and harvested after being induced with isopropyl-β-D-1-thiogalactopyranoside and grown for 3 hours at 37°C. Proteins were then purified by ammonium sulfate cuts, anion-exchange chromatography, and reversed-phase HPLC. The purified protein was lyophilized and stored at -20°C.

Lyophilized protein was dissolved in 20 mM phosphate buffer, 100 mM NaCl (pH 6.5) and filtered using a 0.2 μm filter to remove large molecular weight aggregates. LPS was added to 0, 0.1, 1 or 5 mg/ml. Two-dimensional proton-nitrogen correlation (HSQC) spectra were acquired on a Varian Unity INOVA 600 MHz spectrometer at the Weill Cornell NMR Core Facility equipped with a cryogenic probe at a sample temperature of 10°C, as previously reported.

**Quantitative polymerase chain reaction (qPCR).** The procedure for qPCR has been described elsewhere[^5^](#_ENREF_5). Briefly, total mRNA was extracted from the BV2 microglia cells using RNeasy mini kit (Qiagen, Germantown, MD) and reverse-transcribed using SuperScript VILO cDNA synthesis kit (Life Technologies). The levels of mRNA expressions were determined by StepOnePlus real-time PCR system (Applied Biosystems,Carlsbad, CA) using Taq-Man Fast Advanced Master Mix (Life Technologies) according to manufacturer’s instructions with following gene-specific primers (Life Technologies); IL-1β (Mm00434228_m1), TNFα (Mm00443258_m1), IL-6 (Mm00446190_m1), and β-actin (Mm00607939_s1). Relative mRNA levels were calculated according to the 2-exp (ΔΔCt) method. All ΔCT values were normalized to β-actin.

**Supplementary References**

1 Gath, J. *et al.* Solid-state NMR sequential assignments of alpha-synuclein. *Biomolecular NMR assignments* **6**, 51-55, doi:10.1007/s12104-011-9324-3 (2012).

2 Comellas, G. *et al.* Structured regions of alpha-synuclein fibrils include the early-onset Parkinson's disease mutation sites. *Journal of molecular biology* **411**, 881-895, doi:10.1016/j.jmb.2011.06.026 (2011).

3 Eliezer, D., Kutluay, E., Bussell, R., Jr. & Browne, G. Conformational properties of alpha-synuclein in its free and lipid-associated states. *Journal of molecular biology* **307**, 1061-1073, doi:10.1006/jmbi.2001.4538 (2001).

4 Dikiy, I. & Eliezer, D. N-terminal acetylation stabilizes N-terminal helicity in lipid- and micelle-bound alpha-synuclein and increases its affinity for physiological membranes. *The Journal of biological chemistry* **289**, 3652-3665, doi:10.1074/jbc.M113.512459 (2014).

5 Kim, C. *et al.* Hypoestoxide reduces neuroinflammation and alpha-synuclein accumulation in a mouse model of Parkinson's disease. *J Neuroinflammation* **12**, 236, doi:10.1186/s12974-015-0455-9 (2015).
